# Therapeutic and diagnostic applications of nanoparticles in the management of COVID-19: a comprehensive overview

**DOI:** 10.1186/s12985-022-01935-7

**Published:** 2022-12-03

**Authors:** Omid Gholizadeh, Saman Yasamineh, Parya Amini, Hamed Afkhami, Abbasali Delarampour, Sama Akbarzadeh, Rasool Karimi Matloub, Mahlagha Zahedi, Parastoo Hosseini, Mehrnaz Hajiesmaeili, Vahdat Poortahmasebi

**Affiliations:** 1 Department of Bacteriology and Virology, Faculty of Medical Sciences, Tabriz University of Medical Sciences, Tabriz, Iran; 2 Research Center for Clinical Virology, Tehran University of Medical Sciences, Tehran, Iran; 3 Young Researchers and Elite Club, Tabriz Branch, Islamic Azad University, Tabriz, Iran; 4 Department of Microbiology, School of Medicine, Yasuj University of Medical Sciences, Yasuj, Iran; 5 Department of Medical Microbiology, Faculty of Medicine, Shahed University of Medical Science, Tehran, Iran; 6 Microbiology Department, School of Medicine, Zahedan University of Medical Sciences, Zahedan, Iran; 7 Department of Animal Biology, Faculty of Natural Science, University of Tabriz, Tabriz, Iran; 8 Students Research Committee, Arak University of Medical Sciences, Arak, Iran; 9 Department of Pathology, Faculty of Medicine, Shahid Sadoughi University of Medical Sciences, Yazd, Iran; 10 Department of Virology, School of Public Health, Tehran University of Medical Sciences, Tehran, Iran; 11 Department of Microbiology, Faculty of Medicine, Babol University of Medical Sciences, Babol, Iran

**Keywords:** Nanoparticle, COVID-19, AgNPs, Vaccine, Diagnose, Treatment

## Abstract

In December 2019, Coronavirus Disease 2019 (COVID-19) was reported in Wuhan, China. Comprehensive strategies for quick identification, prevention, control, and remedy of COVID-19 have been implemented until today. Advances in various nanoparticle-based technologies, including organic and inorganic nanoparticles, have created new perspectives in this field. These materials were extensively used to control COVID-19 because of their specific attribution to preparing antiviral face masks, various safety sensors, etc. In this review, the most current nanoparticle-based technologies, applications, and achievements against the coronavirus were summarized and highlighted. This paper also offers nanoparticle preventive, diagnostic, and treatment options to combat this pandemic.

**Graphical Abstract:**

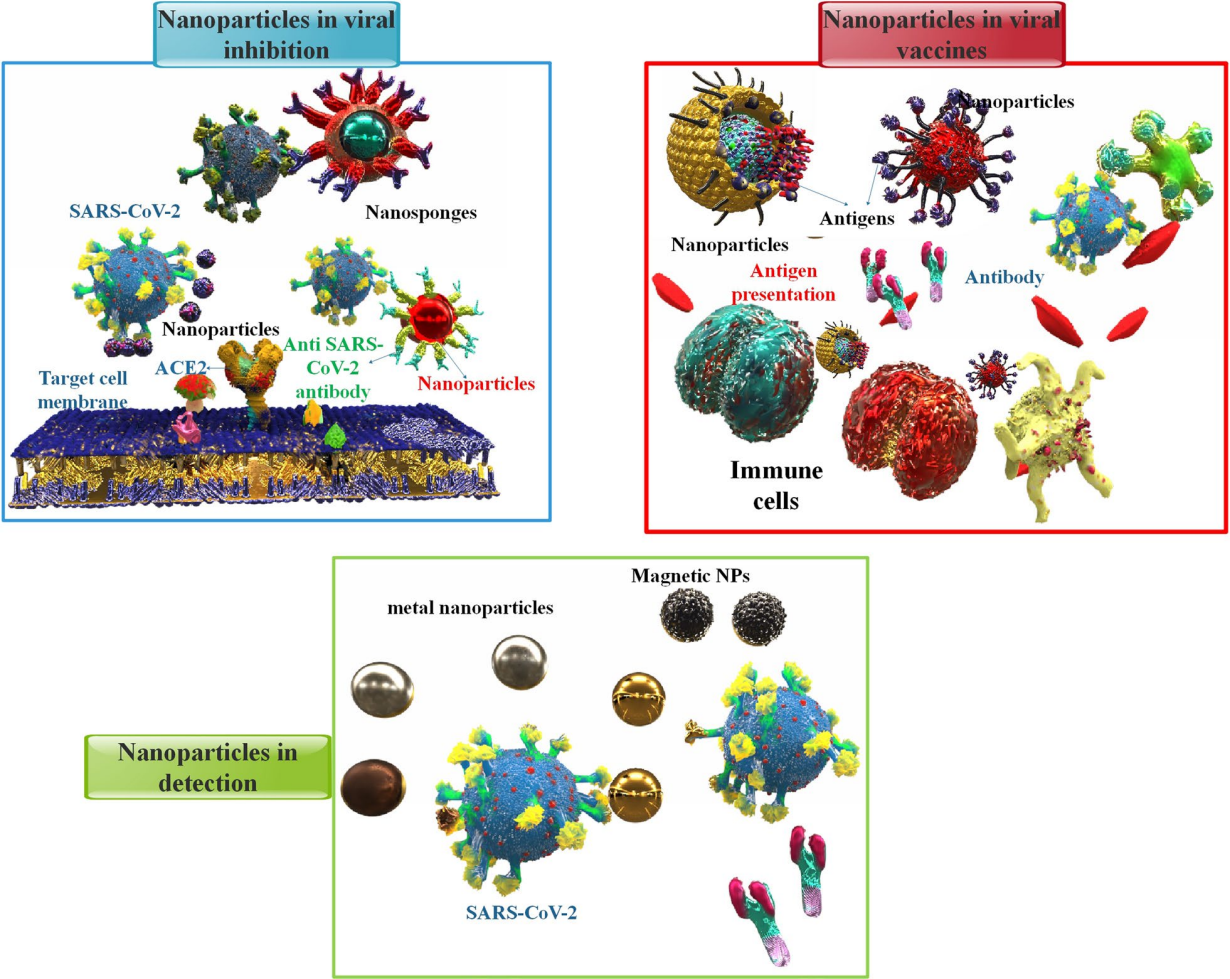

## Introduction

**Fig. 1:**
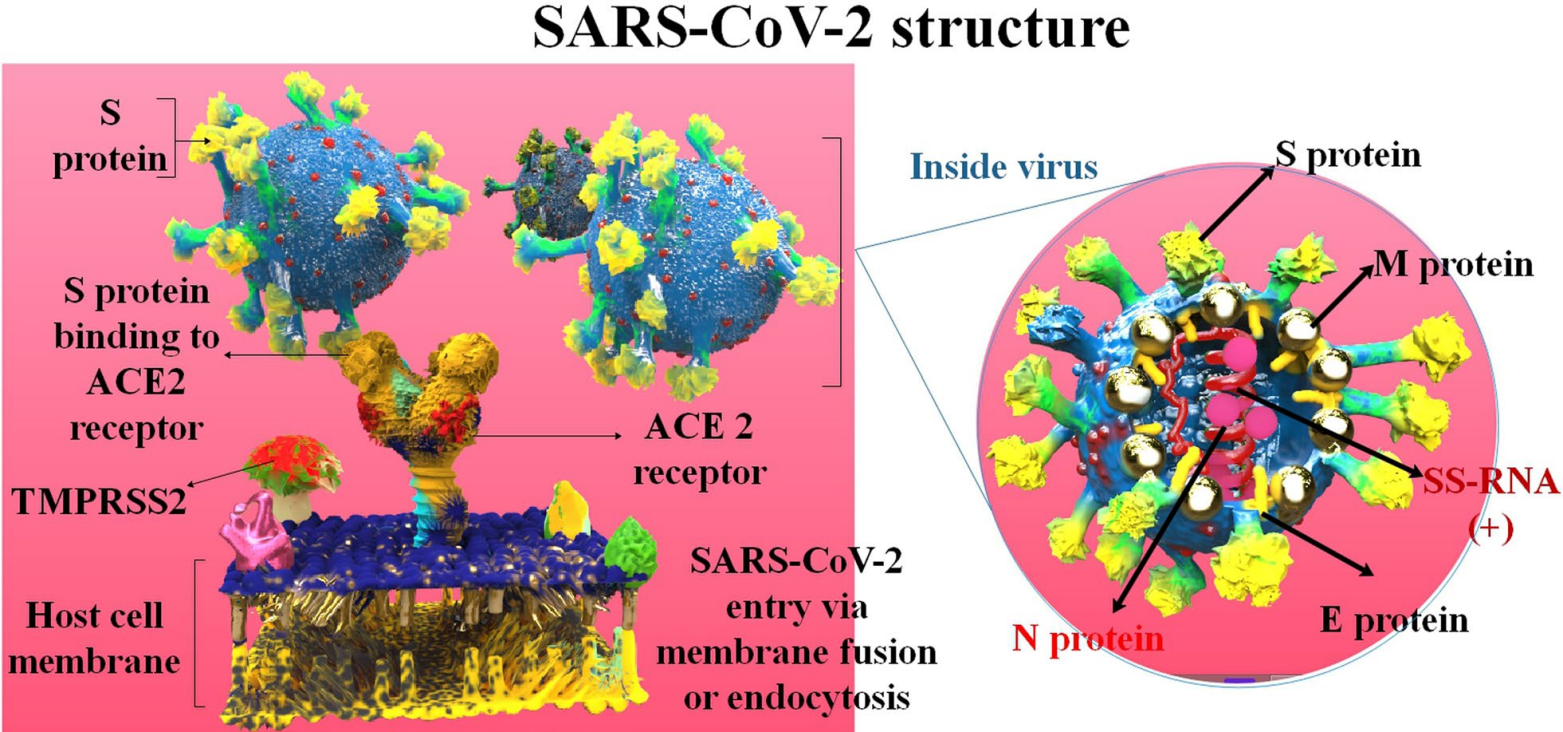
The structure of the SARS-CoV-2 and how it binds to the target cell receptor (ACE2 and TMPRSS2).

Phylogenetically, coronaviruses (CoVs) are divided into four groups: Alpha-, Beta-, Gamma-, and Delta-CoVs. The two primary types of human CoVs are alpha and beta-CoVs. The coronavirus disease 2019 (COVID-19) pandemic is caused by the SARS-CoV-2 (severe acute respiratory syndrome coronavirus), which belongs to the beta-CoV family [1]. About 636 million people have been infected with this SARS-CoV-2, and about 6.5 million patients have died due to COVID-19 by November 2022 [2]. SARS-CoV-2 is enveloped, positive-sense, single-stranded RNA (+ ss-RNA). This virus has many crucial genes that can be per se targeted for viral inhibition platforms advance. These contain the genes encoding the spike protein, RNA replicase, 3 C-like protease, papain-like protease (PLPro, the protease domain of nsp3), and other essential enzymes [3, 4]. Furthermore, SARS-CoV-2 has four various structural proteins, N (nucleocapsid), M (membrane), E (envelope), and S (spike). Practically, the S protein is accountable for connection with the target cell receptors (including angiotensin-converting enzyme 2 (ACE2)), which is involved in the entrance of the SARS-CoV-2 into the host cells [5] (Fig. 1). S proteins are cleaved to produce S1 and S2 segments. The S1 protein contains the N-terminal domain (NTD) and receptor-binding domain (RBD) domains, while the S2 protein promulgates membrane fusion. The RBD is identified via several strongly neutralizing monoclonal, serum antibodies, and protein-based suppressors [6]. Mutations in the SARS-CoV-2 happen automatically in replication. Thousands of cumulative SARS-CoV-2 mutations have occurred since the appearance of COVID-19. As new conversions continue to appear, intrinsically, novel mutants are more and more detected. The majority of SARS-CoV-2 RNA changes do not significantly alter the frequency and severity of the virus or the course of the illness. Mutations in the S protein result in vaccine evasion as the condition is associated with protein site and affinity [7]. There have been several variants of SARS-CoV-2, one of which is the Omicron variant (B.1.1.529). The Omicron variant is the most mutated COVID-19 form, and it has a high prevalence and immune escape capability, which have increased worldwide concerns. Due to its increased prevalence, Omicron has quickly replaced the Delta variant as the dominant variant in different countries [8]. The delta variant was initially identified in India in December 2020 [9]. The lethality rate of this viral infection is about 2–3% and still, it is highly contagious. The symptoms include headaches, sore throat, a runny nose, fever, and cough, which are common symptoms of the omicron variant, along with fatigue and lethargy [10]. Some other signs could be manifested from one patient to another, like loss of taste and smell or shortness of breath. However, according to the new investigation, signs of the Delta variant are getting milder, so patients might think they have a heavy cold [11, 12]. It takes about two days to two weeks from the first exposure to CoVs until the symptoms start. Although, there are many reports of positive asymptomatic cases worldwide, with a proportion of 10.1–23.0% of all tested cases [13]. Due to vaccination, the severity of the symptoms of the disease has decreased [14].

Nanoparticles (NPs) are minuscule materials with dimensions of between 1 and 100 nanometers. The NPs range from organic materials like gold, silicon dioxide, and graphene to inorganic materials like liposomes, micelles, proteins/peptides, and dendrimers. Because of their exceptional ability to absorb biomolecules, NPs are very valuable in healthcare projects [15, 16]. The use of NPs in drug delivery has a lot of benefits [17]. These include the following: (1) Improving the solvability of some medicines; (2) Continuous unleashing of medicine for a long time [18]; (3) Reducing some medicines’ adverse effects. (4) Aiming at particular cells so that the specific drug is not destroyed and can reach the target cells [19]. With remarkable efficiency, NPs can absorb proteins, therapeutic molecules, and a wide range of chemical substances. However, nanostructure toxicity is the primary concern of researchers. Therefore, reducing their toxicity and improving their bioavailability will increase NPs’ effectiveness as diagnostic and therapeutic tools for COVID-19 coping techniques are constructed utilizing NPs, concentrating on the interactions between NPs and subsequently, the absorption of substances like proteins and drugs [15, 16]. Table 1 summarized a few of the NPs’ contributions to the COVID-19 defense. The fight against CoV infection will be assisted by a substantial contribution from the nanotechnology community. Nano-antimicrobials are essential for the prevention, detection, and treatment of viral infections. In Table 2, some nanotechnology applications for COVID-19 prevention and treatment were reported. Whereas necessary resources are committed to hindrance, diagnosis, and therapy, more significant efforts may be created to contain the spread of unwellness. As a result, using NPs on surfaces can prevent CoV transmission in the environment. This approach to nanoparticle technology can provide a relative answer to today’s society and future problems [20]. For example, in the future, nanovaccines that carry SARS-CoV-2 specific antigens will have an enhancing function in developing or improving vaccination results versus SARS-CoV-2 infection. Currently, more than 26 nanovaccine candidates have progressed into clinical trials, with about 60 more in preclinical advance [21]. In addition, NPs as delivery systems can enhance the efficiency of medication transfer against the SARS-CoV-2 infection as therapeutic methods [22]. Moreover, NPs can be injected by several forms of delivery, such as but not restricted to intranasal, intravenous administration, and topical administration. NPs in COVID-19 diagnostic testing allows for on-site testing, which eliminates the typical waiting period and enables fast diagnosis. Given the time-sensitive nature and significance of COVID-19 testing, the performance of NPs to produce point-of-care diagnostic methods may prove beneficial [23–25]. In this article, many nanotechnology applications have been discussed, including the development of vaccines, therapeutic drugs, and detection techniques based on NPs that are now undergoing clinical trials and have the potential to replace existing treatments for SARS-CoV-2 infection.

**Table 1:** Role of different NPs against COVID-19

NPs	Description	Ref.
Lipid nanoparticles (LNPs)	High nucleic acid (siRNA or mRNA) encapsulation rate and enhanced transfection effectiveness resulted in viral load suppression in the lungs The ionizable lipids are close-charged at physiological pH, making them an excellent vehicle for nucleic acid treatments Protecting mRNAs from ribonucleases and delivering their intact form to the target site Lower cytotoxicity and immunogenicity Compared to bilayer liposomes, LNPs exhibit increased cargo stability and cellular penetration Easy manufacturing technique, small size, and serum stability	[26] [27, 28] [29] [30–32] [29] [27, 28]
Gold nanoparticles (AuNPs)	Used for sterilizing and employed in PPE Fast detection of particular COVID-19 viral antigens Common antigen carriers are utilized in immunization and vaccination, increasing vaccination effectiveness by stimulating antigen-presenting cells and maintaining controlled antigen release	[33–36]
Silver nanoparticles (AgNPs)	Silver is effective against all infections, including bacteria, fungi, and viruses. Developing extremely sensitive biosensors based on AgNPs Direct virucidal activity by adhering to the virus particles and blocking their connection to host cells AgNPs attach to the viral genome, resulting in viral replication suppression and the release of progeny virions Producing free radicals and ROS, causing apoptosis and therefore preventing viral infection Triggering an immune response and avoiding COVID-19 inflammation and fibrosis by decreasing the amounts of cytokines and cytokine-mediated inflammation Inhibiting cytokine storm, inflammatory signaling, pulmonary insufficiency reduction, and regulation of the EMT signaling cascade Therapeutic serum and procedures based on NagC to prevent viral and bacterial respiratory infections from spreading Preventing secondary microbial infections after COVID-19 infection	[37] [38] [39] [40] [41] [42] [38] [43] [38]
Polymeric nanoparticles (PNs)	Increasing oral delivery of drugs with improved stability within the digestive tract, shielding encapsulated substances, modulating chemical properties, drug release, and antigen carriers The most frequently utilized NPs for encapsulating chloroquine	[44–46]
magnetic nanoparticles	Rapid detection of virus Practical, automated, and high-throughput alternatives to centrifugation	[47–50]
Zinc nanoparticles	Inhibiting viral reproduction, reducing inflammation, and improving the immune system in specific sites	[51]
Dendrimer nanoparticles (DNs)	Dendrimers have been developed for transdermal, oral, ocular, and pulmonary drug delivery. Limited clinical applications because of its toxicity Low polydispersity and biocompatibility	[16, 52]

**Table 2:** Nanotechnology application for COVID-19 prevention and treatment

	Nanoparticles			
Prevention	Facemasks	Providing wearing comfort, increasing protection against small particles, producing bio-reversible masks	Silver and copper-based nanostructures, biosensors, carbon nanotubes	[53, 54]
	Disinfection of surfaces and PPE	Long-lasting surface disinfectors, self-sterilization, a spectrum of antimicrobial actions (antibacterial, antifungal, and antiviral), bio-safety	Metallic NPs, especially silver	[55]
	Vaccine	Protecting the loaded antigen, passing tissue barriers, multiple targeting, manageable size and surface features, immune stimulatory properties, controllable drug release	Vaccine adjuvant NPs, Nano carriers	[36, 56]
Therapeutic	Detection	Rapid and early-stage detection with excellent efficiency by anti-spike antibodies, minimum contamination, and increased sensitivity.	Gold and graphene-based NPs	[57]
	Antivirals	Improving low solubility and bioavailability, reducing systemic toxicity, crossing biological barriers, increasing short half-life, controlling drug delivery	Nanocarriers such as polymeric NPs, Nanomedicine for combination drug therapy	[46, 58]

## Nanoparticles as a preventive strategy in COVID-19

**Fig. 2:**
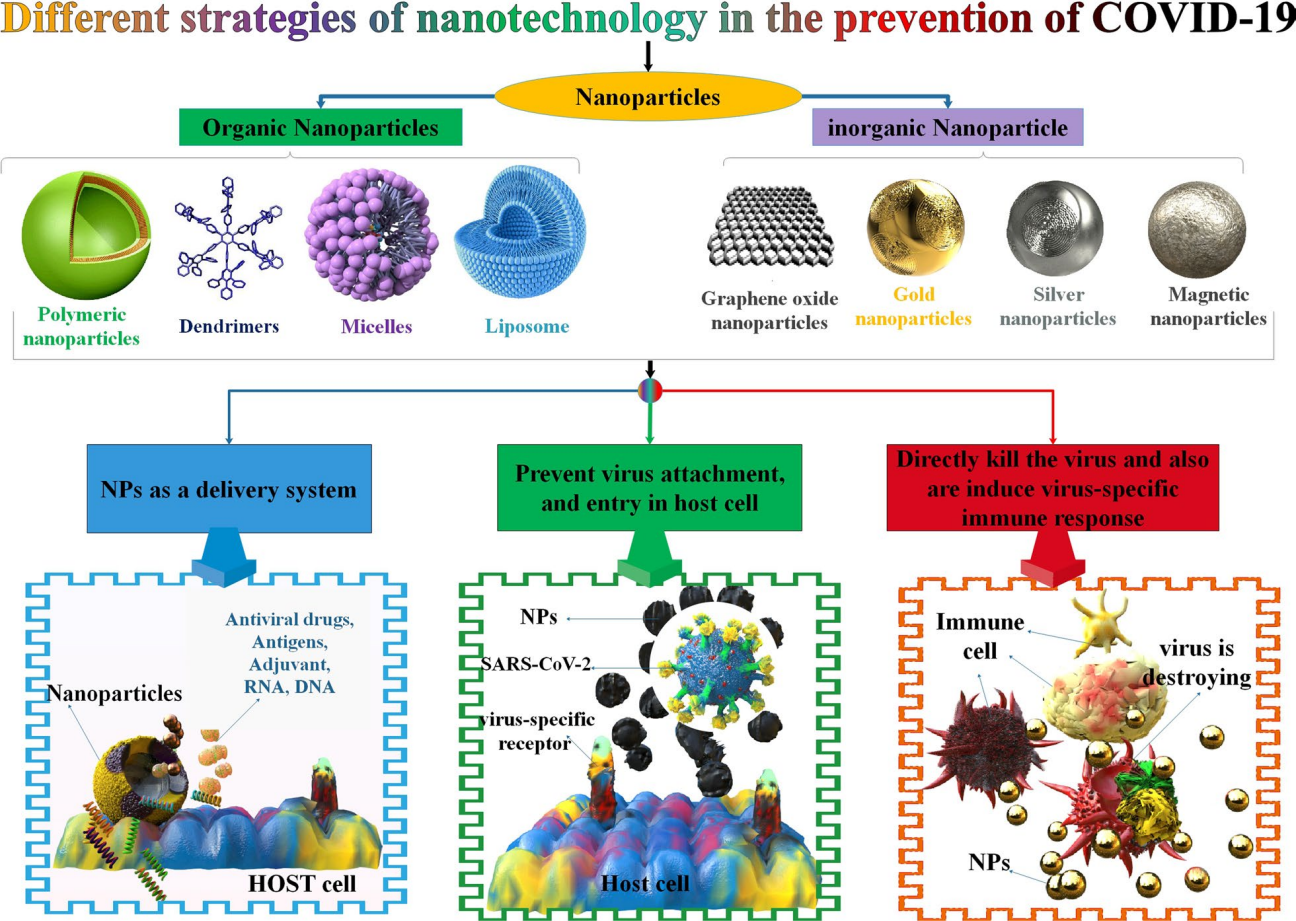
A different strategy of nanotechnology in preventing COVID-19 by several types of NPs. These NPs, include organic NPs (such as polymeric NPs, nanocapsules, nanospheres, liposomes, micelles, and dendrimers) and inorganic NPs (different metallic NPs, and GONPs).

NPs in biomedicine include the completion of separate elements with nanoscale properties (1–100 nm) that permit the creation of a modified two-molecule device that can identify specific types of cells, viruses, bacteria, and fungi [59–61]. Nanoscale data may be transferred to macromolecular data using these approaches. As a result of their unique characteristics, such as their small size and flexibility, NPs are frequently able to direct them to the most suitable drugs and make them safer. In recent years, there have been several attempts to discover alternative techniques to improve nanoparticle impacts on specific viruses that cause respiratory disease, and the outcomes have been encouraging [62]. In Table 3, you can see the function of several NPs in viral respiratory infections. NPs stabilize and unharness the active elements of the immunizing agent within the body. They will even be used as vaccine transmitters [63, 64]. One of the best medicinal therapies for boosting the immune system’s defenses against infectious diseases is vaccination. Meanwhile, since NPs have been shown to possess immune-stimulating features, much attention has been drawn to the production of therapeutic agents or nano-based vaccines against different types of CoVs. Nanovaccines are involved in protecting the host cell and also the immune and immunologic response. Meanwhile, antiviral agents are concerned with preventing infectious agent attachment, cell entry, and systemic infection [65]. Lipid NPs (LNPs) differ from bilayered liposomes because they offer greater cargo stability with a rigid structure and assist in better cellular penetration [29]. LNPs have a high nucleic acid encapsulation rate and enhanced transfection effectiveness. Furthermore, LNPs have less immunogenicity and cytotoxicity than liposomes, which makes them more effective delivery means for nucleic acid-based treatments [30–32] (Fig. 2). In a study, researchers showed that CRISPR-Cas13d provides a wide range of viral inhibition to suppress many COVID-19 virus variants and various human CoV strains with more than a 99% decrease in the viral titer. Cas13d-interceded CoV prevention is associated with the CRISPR RNA (crRNA) cellular spatial co-localization with Cas13d and target viral RNA. Utilizing LNP interceded RNA transfer shows that the Cas13d approach can successfully remedy infection from several variants of CoV, such as the Omicron variant, in human early airway epithelium air-liquid interface (ALI) cultures [66]. Chitosan is an abundant organic polysaccharide, which can be partly acquired via chemical modification of animal or fungal-origin substances. Chitosan and its derivatives have been demonstrated to show immediate antiviral function, to be beneficial vaccine adjuvants, and to possess the capability of anti-COVID-19 activity [67]. For instance, the polyphenolic extracts were mixed with bovine serum albumin (BSA) moieties before being coupled with chitosan, a mucoadhesion polymer, to form capsules. Furthermore, entrapping silymarin demonstrated viral inhibition function in vitro SARS-CoV-2 infection experiment. It can be briefed that the muco-inhalable transfer method packed via silymarin can be utilized to overcome inflammation stimulated via oleic acid and to inhibit SARS-CoV-2 infection [68]. According to research, Molecularly Imprinted Polymers (MIPs), which may join a portion of the COVID-19 virus S protein electively, are essential for the growth of plastic antibodies. Molecular printing displays a promising and marvelous technology for preparing MIPs determined via particular diagnosis capabilities for a target molecule. Due to these features, MIPs can be considered artificially made antibodies attained via a patterning procedure. Following bioinformatics investigation, imprinted NPs were prepared via the inverse microemulsion polymerization method, and their capability to inhibit the interplay among ACE2 and the RBD of the COVID-19 virus was researched. Importantly, the generated artificial antibodies can remarkably suppress virus reproduction in Vero cell culture, offering their capability in the therapy, inhibition, and detection of COVID-19 [69]. In a different study, scientists created two NP-based drug delivery systems: polymeric NP and nano micelles based on azithromycin and hydroxychloroquine. The results demonstrated the effectiveness of the processes used to produce the nanomicelles and NP. The attributes demonstrated an NP with a spherical form and a mean dimension of 390 nm a nano micelle and with a spherical form and a mean dimension of 602 nm. The nano micelles were more effective (about 70%) versus COVID-19 than the NPs. None of the NPs demonstrated cytotoxic efficacy on FGH cells, even in extreme amounts, confirming the lack of side effects of both NP drug delivery systems [70]. The VIPER (virus-inspired polymer for endosomal release) block copolymer has been introduced as an encouraging delivery method for both plasmid DNA and short interfering RNA (siRNA). After determining siRNA/VIPER polyplexes, the acting and harmlessness profiles were corroborated in a lung epithelial cell line. This method effectively suppressed COVID-19 infection prevalence. This NP demonstrated the potential of local siRNA transfer as an effective antiviral therapy in the lung by carrying siRNA to lung epithelial cells and interfering with substantial downregulation of viral replication both in vitro and ex vivo without producing toxic or immunogenic side effects [71].

**Table 3:** Function of several nanoparticles in viral respiratory infections

Type of the Viral Infection	Type of the Nanoparticle	Description		Ref
Influenza	TMC NPs	To successfully prevent this infection, purified HA2 and NP recombinant proteins were encapsulated in TMC NPs		[72]
H1N1	PS-GAMP	PS-GAMP significantly boosted H1N1 vaccine-induced humoral and cytotoxic T-cell immune responses in mice by imitating the primary stage of illness without causing further inflammation		[73]
H1N1	PEGylated ZnO-NPs	Exposure to the virus and bare ZnO-NPs at the highest non-toxic concentrations may result in a drop in viral titer		[74]
H3N2	AgNPs	The inhalation route used to inject AgNPs increases survival in H3N2-infected mice		[75]
H1N1	SeNPs	SeNPs inhibit lung damage in H1N1-infected mice and repress interactivity among virus and target cells		[76]
H1N1	IO-NPs	These NPs prevent the virus from connecting to target cells in vitro		[77]
RSV	AgNPs	Curcumin-modified AgNPs have more significant antiviral activity and a more effective inhibitory impact against respiratory syncytial virus (RSV) infection		[78]

Furthermore, investigators in immunology frequently add NPs, recognized as nano-adjuvants, to vaccines. NPs used as vaccine adjuvants are usually categorized as inorganic NPs (such as AgNP, AuNP, Copper nanoparticle (CuNP), and Fe2O3 NPs) or organic (such as polymeric, lipid base NPs, and cell base NPs). Since different NPs can function as adjuvants and possible delivery systems of vaccine antigens, they have attracted the consideration of investigators [79, 80]. The dimensions of NPs assist in simplifying absorption via antigen-presenting cells, resulting in efficient antigen diagnosis and exposure. The alteration of the covers of NPs with various targeting moieties allows the transfers of antigens to particular receptors on the cell outside and elicits elective and special immune reactions [81]. In addition, viral inhibition pathways contain oxidative damage and binding protein suppression to disable a wide range of viruses successfully. Remarkably, the tremendous reactive surface area to volume proportion and unique chemical attributes of metal or metal oxide NPs allows their substantial neutralization of viruses. Reactive oxygen species (ROS) oxidation, destructive replacement connections with key viral structures, and inhibition of virus-cell receptor connection are three ways that NPs carry out their virucidal activity [82]. Silver NPs (AgNPs) have engaged in antiviral and immunomodulatory functions. These NPs are presented to strongly prevent the entrance of the SARS-CoV-2 virus into the target cells. In addition, AgNPs may even decrease the complications of COVID-19 via the inhibition of cytokines, MAPKs, and NF-κB signaling [38]. Moreover, these metal NPs decreased the amounts of inflammatory cytokines, including interleukin (IL)-1 b, IL-6, IL-17, transforming growth factor-beta (TGF-β), and tumor necrosis factor-alpha (TNF-α) in preclinical animal examples, leading to decreased inflammation and reduced fibrotic cascade [83]. The capacity of AuNPs functionalized with several groups, such as 3-mercapto-ethyl sulfonate (Mes), mercapto undecanol sulfonic acid (Mus), octane thiol (Ot), and a novel peptide, to decrease COVID-19 was investigated using molecular dynamics (MD) simulations. Functionalized AuNPs have significant efficacy on the RBD and potently interrelate with this protein of the COVID-19 virus. Furthermore, the AuNP functionalized via novel peptide formations a more constant complex with RBD in contrast with ACE2. Several evaluations vouch that the produced AuNPs can be well selected for antiviral factors versus SARS-CoV-2 infection [84].

Using NPs in antiviral coverings on surfaces is a significant issue in reducing infection transmission in the environment [20]. Much emphasis has been paid to the antibacterial and antiviral characteristics of the two-dimensional nanomaterials’ graphene and graphene oxide (GO). It is possible to identify viral proteins using graphene sheets attached to antibodies [85]. When combined with heat or light-mediated inactivation, functionalized graphene might be employed as a disinfectant due to its capacity to trap viruses. Using an antibody against the virus’ spike protein, researchers created graphene-based field-effect transistors (FETs) to detect COVID-19 viral load in clinical samples. A FET-based biosensor is used with a cultivated virus, antigen protein, and nasopharyngeal swab samples from ill individuals to detect SARS-CoV-2 [86]. NPs are becoming increasingly popular as a way of reducing viral transmission across the world. On the other hand, the widespread availability of single-use masks represents a danger to the environment. As a result, using graphene as a breathable protective layer in face masks decreases the risk of transmission while allowing the face masks to be reused [87]. NPs are used as polymeric moieties in filters, fabric, and facemasks to inactivate viruses [53]. Water penetration of the facemask’s surface affects its maintenance. When the surface is hydrophobic and dry, germs cannot enter the protective layers. Therefore, photocatalysis or heat may recycle this face mask [88–90]. Under sunlight, the high surface temperature of the face mask may effectively sterilize surface diseases. Viruses may be denatured after 30 min of contact with graphene at 56 degrees Celsius. A range of sophisticated NPs has been identified that can be utilized to create multipurpose antiviral facemasks [91]. As a result of their high surface volume ratio, small pore size, versatility, acid and base resistance, excellent mechanical properties, and high light-to-heat conversion efficiency, carbon nanotubes (CNTs) have become increasingly employed in biology and health science in recent years [92–94]. For COVID-19, CNTs offer special qualities including ample storage capacity and surface area, excellent biocompatibility, high penetration of biological barriers, tailored biomolecule modification potency, multi-energy surface/tube chemical functional group capabilities, and high bioabsorption rate. CNT can also be used as a filtration agent, a diagnostic system, a viral inactivation agent, and medication delivery [95, 96]. Another possible use of CNT against COVID-19 is the manufacture of N95 facemasks, which inhibit viral transmission from person to person [54, 97–100]. NPs have also been effectively utilized as antivirals. Other materials that might be utilized for sterilization include silver, iron oxide, gold, and titanium dioxide (TiO) NPs, which exhibit antiviral properties comparable to those of polyphenylene ethynylene (PPE) [33–35]. Drug resistance is also one of the world’s current issues. NPs have antibacterial and antiseptic properties, so they can be used to reduce drug resistance. In addition, microorganisms can mutate against traditional antimicrobials and are likely to grow again. In contrast to conventional antimicrobials, NPs are present since the use of NPs is expected to reduce mutations and the resistance of microorganisms to these antimicrobial NPs, thus decreasing the regrowth of microorganisms [101].

Because of their strong antimicrobial properties, AgNPs offer a unique way to limit SARS-CoV-2 while preventing secondary microbial infections. Antiviral effects of AgNPs with a size of 2–15 nm have been demonstrated against SARS-CoV-2. Additional results include cytokine storm inhibition, inflammation signaling decrease, respiratory failure reduction, and epithelial-mesenchymal transition (EMT) signaling cascade control. Furthermore, AgNPs provide a variety of options for developing extremely sensitive biosensors. It is strongly believed that AgNPs will play a critical role in defeating the COVID-19 pandemic in the not-too-distant future [38]. AgNPs have shown antiviral activity. Social reduction strategies and nonspecific drug therapies have been implemented. Given that this is an emerging virus, studies on NPs are still scarce [102]. Silver is effective against all infections, including bacteria, fungi, and viruses. Because of their unique catalytic, electrical, chemical, and optical characteristics, antimicrobial agents in the health sector, electrical components, sensors, and catalysis are just some of the uses for AgNPs. AgNPs can be applied to some inanimate surfaces to counteract the continuing COVID-19 outbreak. Disinfectants and sanitizers based on aluminum are also used to disinfect inanimate surfaces and hands [37]. The current work is provided as an urgent recommendation for associated medicinal researchers to investigate the possible usage of AgNPs as a candidate for likely inhibition of COVID-19. According to the studies reviewed above, certain AgNPs have been utilized to suppress fungi, bacteria, and viruses in vitro and in vivo [103]. Researchers have employed this nanoscale material to stop COVID-19 [104]. On the other hand, other studies argue that AgNPs should be used in clinical trials or even in clinical practice for various therapies. In addition, several evaluations claim that silver was a beneficial substance in ancient medicine for multiple treatments. It is proposed that (by associated scientists) at least the potential of AgNPs, as well as numerous non-hazardous nano metals and nano metal oxides (at particular doses), may be regarded as possibilities for suppression of SARS-CoV-2 [103].

The goal of the current research was to create a broad-spectrum disinfectant with high antibacterial AgNPs for surgical mask shielding that would be accessible and simple to use for medical personnel and the general public. As a result, a simple electrochemical etching technique was used to make AgNPs. The AgNPs initially had a spherical shape and were strongly monodispersed [105]. According to experts, SARS-CoV-2 may live for over 14 days at 4 °C, indicating the virus’s long-term potential to transmit via contact with infected surfaces. Despite its remarkable capacity to survive, this virus is inactivated and eradicated by various disinfectants, including those based on alcohol formulations [106]. As a result, they developed a novel disinfectant for surgical mask disinfection and shielding based on a 45% ethanol solution containing protein-destabilizing surfactants, super oxidized water, and triclosan reinforced with AgNPs by using Dey and Engley’s suggested recovery media method [107].

## Nanoparticles as a great blessing

People who are immunocompromised as well as those who have comorbid conditions such diabetes, heart disease, renal disease, liver disease, and other illnesses are at a higher risk of infection. Scientists are working around the clock to develop a new medication or vaccine that will be successful against the deadly virus. The use of NPs and the science of NPs can hope to reduce the threat to human life worldwide. Nanotechnology is seen as the future technology of the twenty-first century, and it is viewed as a major boon to medical research. Nanotechnology comprises two words: technology and the Greek numerical term “nano,“ which means “dwarf.“ As a result, the science and technologies used to manufacture or manipulate particles with a size range of 1 to 100 nanometers are referred to as nanotechnology [108, 109]. Nanobiotechnology is a discipline that combines nanoscience, biotechnology, and the related area of nanomedicine, which focuses on employing nanostructured materials to diagnose, treat, and prevent diseases. Combination medication therapy is another option for treating COVID-19. It has many advantages, including decreasing individual drug doses with fewer side effects, reaching several complementary therapeutic objectives, and lowering the risk of resistance. For instance, nanocarriers are very helpful for conveying numerous medications with various physicochemical qualities, enabling combination treatments to reach their full potential [110, 111]. Given the lack of a proven therapy for COVID-19, focusing on a less researched area, such as the cytokine storm, also known as cytokine release syndrome (CRS), may be beneficial [112]. A complication seen in many COVID-19 patients with severe disease is systemic inflammatory response syndrome, which may play a key role in the disease’s latency [113]. Cytokines are normally produced when a pathogen is recognized to recruit and regulate the immune response. However, COVID-19 may cause CRS, a disease in which the immune system produces pro-inflammatory cytokines out of control [114–117]. IL-6 and TNF-α are the two main pro-inflammatory cytokines identified at increased levels in COVID-19 individuals with CRS [118–120]. Damage is caused by an increase in IL-6 and TNF, which may result in tissue deterioration, acute respiratory distress syndrome, organ failure, and death [119]. CRS is a life-threatening illness that must be treated if the health of COVID-19 patients is to improve. It can harm organ systems as well as the immune system. As a result, a new approach shows that developing IL-6 and TNF antagonists might be a feasible method for treating CRS and, more broadly, SARS-CoV-2 [121, 122]. Novel therapeutic medicines that directly capture and neutralize cytokines are critical for reducing the excessive immunogenic response of CRS. In clinical trials, many monoclonal antibodies are tested to treat SARS-CoV-2-induced cytokine storm and systemic inflammation. Currently, in phase III clinical trials are Tocilizumab, an anti-IL-6 medicine, and Adalimuumab, an anti-TNF therapy that the Chinese Trial Clinic registry is investigating. Both of these drugs have been recommended as treatments for CRS [123]. It has been demonstrated that cytokine-neutralizing antibodies could be attached to NPs to boost their durability, precision targeting, and retention after local injection [124]. Arthritic joints can be treated using anti-IL-6 antibodies conjugated to an anti-cytokine nanoparticle of cross-linked chitosan and hyaluronic acid (HA) [42]. As IL-6 is immobilized on these NPs, significant macrophage (Mφ) suppression occurs, which might lead to the therapeutic use of NPs for CRS in COVID-19 patients [125]. Cell-membrane-coated NPs are another therapy that may decrease the severity of CRS systemic symptoms. Mφ-membrane-coated NPs have an antigenic shape comparable to Mφs, which may trap and neutralize pro-inflammatory cytokines such as IL-6 and TNF-α [126]. In addition to neutralizing TNF-α and IL-1, Neutrophil-membrane-coated NPs, like Mφ membrane-coated NPs, have anti-inflammatory effects [127]. Because NPs have anti-inflammatory properties, they should be explored in treating CRS, which is a major factor in COVID-19-related mortality. The Food and Drug Administration (FDA) previously authorized iron oxide NPs (IONPs) to treat anemia, and studies have shown that they exhibit antiviral activity in vitro. In order to understand how IONPs (Fe_2_O_3_ and Fe_3_O_4_) interact with the SARS-CoV-2 spike protein receptor-binding domain (S1-RBD), which is crucial for viral attachment to host cell receptors, scientists carried out docking study. Therefore, they advise using FDA-approved-IONPs in COVID-19 therapy research studies [128]. The SARS-CoV-2 infection causes a drop in albumin levels, as well as an increase in NETosis (NETosis is a program for the formation of neutrophil extracellular traps [129]), blood coagulation, and cytokine levels [130]. The study proposes drug-loaded albumin NPs as a treatment agent for severe SARS-CoV-2 patients with poor clinical outcomes. The steroidal ginsenoside saponins PEGylated nanoparticle albumin-bound (PNAB)-Rg6 and PNAB-Rgx365 were utilized to enhance the extended bioactivity of PNAB. Their findings show that administering PNAB-steroidal ginsenoside to SARS-CoV-2 ICU patients’ PBMCs may significantly decrease histone H4 and NETosis-related variables in the plasma, as well as SREBP2-mediated systemic inflammation. Their results indicate that these PNAB-steroidal ginsenoside medicines may be used to treat symptoms like coagulation and cytokine storm that are common in severe SARS-CoV-2 patients [131]. It is important to have novel medications as soon as possible to fight the pandemic. Newly, it has been identified that reusing already available medications could be a good method to formulate an efficient drug for SARS-CoV-2 infection. Though there are many FDA-accepted medications, it has been shown that the anthelmintic medication niclosamide (NIC) has remarkably great capability versus COVID-19 [132]. Sanoj Rejinold et al. used a simple self-assembling method in which Zein NPs were effectively utilized to load NICs. Also, these NPs were next coated with bovine serum albumin (BSA) to improve the medications’ constancy, injectability, and particular properties against the infected cells. The BSA-stabilized Zein-NIC nanohybrid was shown to have particle diameters less than 200 nm, well-colloidal consistency, and continuous medication discharge attributes.A hybrid medicine delivery strategy should be highly beneficial for treating SARS-CoV-2 patients who have high endothelial glycocalyx damage followed by a cytokine storm linked to acute inflammations, as shown by the nanohybrid’s improved medication discharge behavior under serum conditions [133, 134]. According to recent research, NPs may be injected into an infected person’s bloodstream and connect to the CoV receptor by utilizing a cavity, which is intended to transmit electromagnetic (EM) wave propagation back and forth between its walls. Standing waves occur at the cavity’s resonance frequency, which is also the frequency at which the response amplitude is highest. The CoV-infected individual is placed in a room that has been adjusted to resonate with NPs connected to CoV receptors. Virus activity in affected people is reduced due to the heat produced by the resonance frequency of EM waves and NPs connected to the CoV [135]. The researchers have developed a therapeutic serum and procedures based on nano-silver colloids (NagC) to prevent viral and bacterial respiratory infections from spreading. Inhalation was used to administer the compositions. The size of the NagC NPs was shown to have a high sensitivity to dose in the research, with 3–7 nm being the best size. The effective antiviral inhibitory concentration (IC) in respiratory mucus of 10 g/mL was a feasible goal to achieve. The research discovered that IC might be produced in the bronchial tree and alveoli by depositing 0.33 cc of a 30 g/mL NAgC concentration, 5 nm colloidal AgNPs, and delivering inhalation of standard 5 m diameter droplet aerosol (e.g., using off-the-shelf ultrasonic mesh nebulizers). The tissue deposition fractions during oral breathing of 5 m aerosol particles include the pharynx (30%), bronchial tree (30%), and alveoli (30%) (25%) [43], the exhaled portion of NAgC may be given through exhalation via the nose or orally to the nasal cavity via nebulized inhalation.

The scientists explore the potential of achieving an effective minimum inhibitory concentration (MIC) of AgNPs in different respiratory system target sites for suppressing both viral and bacterial respiratory illnesses. Two applications include reducing the frequency of ventilator-associated pneumonia (VAP) in hospital ICUs and controlling local COVID-19 outbreaks via early-stage home treatment. Their main goal is to offer a first-line intervention to prevent viral infection from spreading throughout the respiratory system, giving the immune system more time to respond and reducing the danger of virus aggravation and spread. They propose a model method and computation for achieving an antiviral MIC of AgNPs in various respiratory system locations, based on previously published experimental data on the antiviral effectiveness of colloidal silver, by (a) analyzing the nanoparticle size-dependent required concentration. (b) calculating the necessary aerosol delivery properties. We include deposition fraction losses and the inhalation duration fraction of a normal breathing cycle when determining the required delivery dose [136].

## Therapeutic procedures of the SARS-CoV-2 infection

Mutations in the ongoing outbreak of the SARS-CoV-2 genome make it challenging to produce treatment methods. On the other hand, the high prevalence of this virus has led to the rapid development of an effective treatment method [137]. To combat the SARS-CoV-2 prevalence, several medical, social, and engineering approaches have been suggested. These include ways for treatment, prevention, diagnosis, and prediction [138]. For example, the COVID-19 pandemic has resulted in a massive worldwide public health campaign to decrease the prevalence of the SARS-CoV-2 infection via enhancing hand washing, reducing face touching, wearing masks in public and social distancing [139]. 3D printing for engineering and produced do-it-yourself (DIY) preservative instrumentations are used to quickly democratize and deliver health-associated tools and provide them at a low cost [140]. For COVID-19 prevention, several vaccines have been developed, including the mRNA base vaccine (Pfizer-BioNTech and Moderna) and the inactivated vaccine (Sinopharm), as well as a number of pharmacological strategies, primarily based on the repurposing of drugs such as dexamethasone, Favipiravir, and Remdesivir [141]. In addition, SARS-CoV-2 infection is treated with antibiotics, oxygen therapy, cell therapy, antibody therapy, and convalescent plasma approach, which is an immunotherapy strategy using viral-specific antibodies [1, 142]. Based on reports of the World Health Organization (WHO) on November. 3, 2022, vaccine candidates were in clinical assessment to remedy COVID-19, 120 vaccines were in clinical examination, and 11 vaccines were granted Emergency Use Listing (EUL) by WHO [143, 144]. These vaccines contain inactivated vaccines, nucleic acid vaccines, vector vaccines, and subunit vaccines. Given how frequently inactivated vaccines are used to prevent emerging infectious diseases (EID), and how quickly they can be produced, this approach offers optimism for the development of the COVID-19 vaccine [138, 145]. Nucleic acid vaccines (DNA, RNA) present the genetic substance encoding the antigenic protein for the host to express. The composition and immunological potency of the antigen might be significantly altered by host glycosylation since these proteins will withstand host posttranslational changes.These vaccines for several antigens might be made with equal ease, lowering costs even more. Most common vaccine regimens do not permit this [146, 147]. Viral vectors are a relatively novel vaccine method based on recombinant viruses to carry elected immunogens into the target cell. These vaccines can improve immunogenicity without an adjuvant and stimulate a robust cytotoxic T lymphocyte (CTL) reaction to remove virus-infected cells [148, 149]. Subunit vaccines consist only of the pathogens and antigenic ingredients needed to induce efficient immune responses. A protein subunit that includes a particular production of the virus rather than a fundamental viral particle is utilized to cause immune reactions. Moreover, structure proteins, SARS-CoV-2, contain structural and non-structural proteins [150]. In Table 4, you can observe the specifications of some SARS-CoV-2 vaccines in the advanced clinical development phase.

**Table 4:** Specifications of some SARS-CoV-2 vaccines in the advanced clinical development phase

Vaccine platform	Vaccine	Developer	Number of doses	Timing of doses	Approved	Ref.
Inactivated vaccine	The inactivated COVID-19 vaccine with aluminum hydroxide	Sinovac	2	0,14 days	53 countries	[151]
	Inactivated	Beijing Institute of Biological Products/Sinopharm	2	0,21 days	88 countries	[152]
Non-Replicating Viral Vector	ChAdOx1-S	University of Oxford/AstraZeneca	2	0,28 days	137 countries	[153, 154]
	Adenovirus Type 26 vector	Janssen Pharmaceutical Companies	1 2	0 0,56 days	106 countries	[155]
Protein Subunit	Full-length recombinant COVID-19 virus glycoprotein NP vaccine adjuvanted with Matrix	Novavax	2	0,21 days	32 countries	[156]
	The adjuvanted recombinant protein (RBD-Dimer) expressed in CHO cells	Anhui Zhifei Longcom Biopharmaceutical/Institute of Microbiology, Chinese Academy of Science	2–3	0, 28, 56 days, or 0 + 28 days	3 countries	[157]
RNA based vaccines	LNP-encapsulated mRNA	Moderna/NIAID	2	0,28 days	85 countries	[158]
	3 LNP-mRNAs	BioNTech/Fosun Pharma/Pfizer	2	0,28 days	137 countries	[159]
Virus-like particles	Plant-derived Virus-like particles (VLP) are adjuvanted with AS03	Medicago Inc.	2	0, 21 days	–	[160]

## Inorganic and organic nanoparticles in the COVID-19 vaccine

**Fig. 3:**
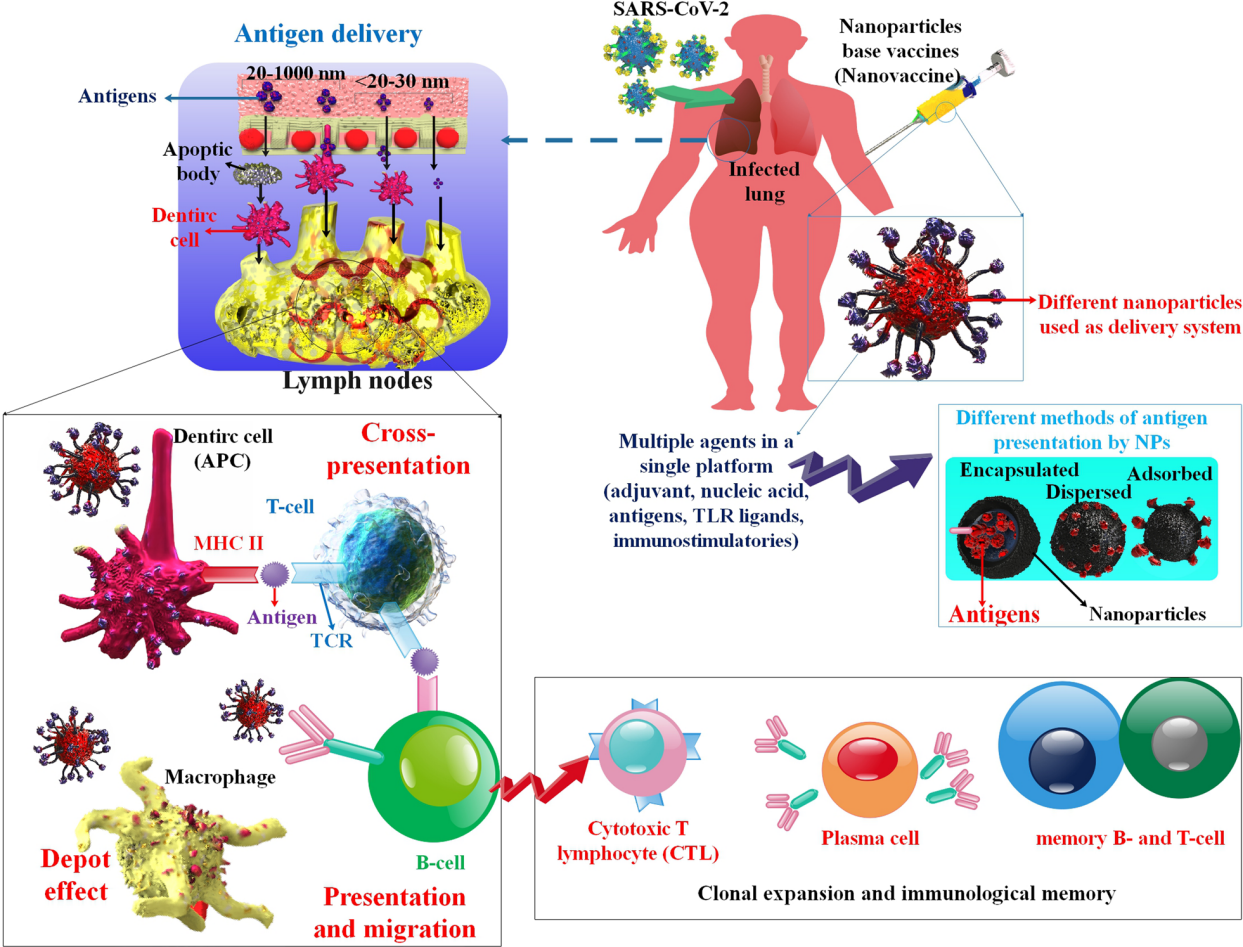
Schematic illustration of the characteristics of the nanovaccines against the SARS-CoV-2 infection. Nanovaccines can enhance cell-intermediated and antibody-intermediated immunity, also immunologic memories over time. Nanovaccines are designed via physical adsorption, chemical conjugation, encapsulation, or physical mixing of antigens with NPs. In addition, antigen delivery by NPs to infiltrated tissue or organ targeting is related to the size of the NPs.

Vaccines have been one of the foremost eminent medical treatments for decreasing infectious unwellness for over a century, and they are estimated to save the lives of millions of individuals every year all across the world. Nevertheless, many diseases are still unpreventable with vaccines, so the world needs newly developed, safe, and effective vaccines [161]. During the COVID-19 pandemic, a wide variety of vaccines were developed expeditiously. Vaccines are produced through different platforms, such as live attenuated vaccines, inactivated, adenovirus-vectored vaccines, recombinant protein, DNA or RNA, and nanoparticle vaccines [162, 163]. Identified antigenic protein components that have been purified are used in today’s vaccinations, making them safer. Self-assembling NPs can be used with structural vaccinology for optimum effectiveness in vaccine antigen rational design [161]. Unwanted side effects have decreased as vaccines have moved from using entire pathogens to just the necessary antigenic epitopes, but associated immune responses have been substantially reduced. Several controlled-release vehicles have been suggested as synthetic vaccines to improve immunogenicity, albeit the emergence of NPs as an especially unique system changed the research path. Particularly, NPs have been shown to be capable of not only delivering a desired vaccine but also of being directed at particular immune cells, loaded with immunostimulatory substances known as adjuvants, or even inducing advantageous immune activation on their own [164, 165]. Oral consumption of peptides and protein drugs ends up with low bioavailability. One of the most promising methods for increasing oral delivery is to mix them with colloidal carriers like polymeric-NPs that are stable within the digestive tract, shield encapsulated substances, and may modulate chemical properties, drug release, and biological behavior. Nanoparticle transport mechanisms via the intestinal tissue layer are addressed and discussed. Enterocytes and/or Mφ cells absorb substances based on their size, and surface features are also discussed. By modeling surface features to optimize access to and transport by Mφ cells [52], as well as identifying surface characteristics specific to human Mφ cells that allow for nanoparticle transcytosis and targeting of Mφ cells, it is demonstrated how to increase their absorption by appropriate cells, i.e. (i) Mφ cells. Although in-vivo studies showed promising results, low bioavailability and a lack of control over the absorbed dose hampered product development. Vaccines are by far the most promising use for NPs given orally [44, 45]. Vaccination is still an excellent way to stimulate protective immune responses against illnesses. Choosing an appropriate carrier that will produce a robust immune response is a crucial problem in producing antibodies and vaccines. Nanoscale particle carriers seem to be particularly promising in this respect. An adjuvant is also used to boost the immune reaction throughout vaccination by using an antigen that has been absorbed or encapsulated by NPs. Gold NPs (AuNPs) are a common antigen carrier utilized in immunization and vaccination, and they’re employed to develop novel vaccines against viral, bacterial, and parasite diseases. AuNPs can be used as adjuvants to increase vaccination effectiveness by stimulating antigen-presenting cells and maintaining controlled antigen release [36]. NPs, roughly the same size as viruses, can enter cells and produce antigens from nucleic acids (mRNA and DNA vaccines) or transport antigens directly to immune cells (subunit vaccines). As part of the vaccine development process, the viral antigen could be encapsulated in a nanosystem or connected to nanocarrier surfaces for adjuvant administration. LNPs and polymeric-NPs are widely used as antigen carriers. Adjusting the NPs’ size, structure, and charge may increase the vaccine’s effectiveness [166] (Fig. 3). There are nanotechnology-based formulations used by Pfizer/BioNTech and Moderna.BNT162b2 (Pfizer-BioNTech) and mRNA-1273 (Moderna) obtained emergency FDA use authorization (EUA) in the United States, providing sufferers with renewed optimism that their COVID-19 would be better treated shortly. In Table 4, you can observe the specifications of some SARS-CoV-2 vaccines in the advanced clinical development phase. The mRNA is delivered to the target location in its intact form by NPs, which also help to shield it from ribonucleases, increasing stability. The remarkable efficacy of these two mRNA-based vaccines is due to the unique nanocarrier, “LNPs,“ which reached 95% efficiency in phase III clinical trials. Compared to bilayered liposomes, LNPs are more stable, have a harder structure, and are better at penetrating cells. For nanotechnology, the EUA is a watershed moment. It is via these two vaccinations that COVID-19 has been brought under control, proving the usefulness of nanomedicines in solving global health issues [29]. LNPs allow siRNA or mRNA to enter host cells more effectively. LNPs are frequently employed to carry antigen-encoding mRNA and to host viruses including influenza, rabies, human immunodeficiency virus (HIV), and the cytomegalovirus (CMV) [167, 168]. Because of their easy manufacturing technique, small size, and serum stability, LNPs are chosen over other carriers for delivering nucleic acids into cells. As a result of the negative charge of biological membranes and nucleic acids, mRNA delivery is challenging. In LNPs, the ionizable lipids are positively charged at physiological pH, making them an excellent vehicle for nucleic acid treatments. In particular, ionizable lipids should be engineered to be positively charged at acidic pH through the manufacture of LNPs so that the electrostatic interplays with the nucleic acids could be amplified (great nucleic acids amount is equal to grate loading efficiency). Afterward, the ionizable lipids should change to nearly neutral under physiological situations (pH 7.4) to inhibit quick separating via immune cells in the systemic circulation (through thr Kupffer cells in the liver and splenic Mφ, positive NPs are promptly sorted). Effective intracellular transport and escape from endosomes are made possible by the ionization caused by the endosomal acidity (pH = 4.5) [27, 28, 169]. Because the NPs may display several antigens simultaneously, vaccinations can trigger a much larger range of immune responses. For example, human papillomavirus (HPV) and hepatitis B virus (HBV) vaccines use virus-like particle vaccines that provide significant long-term protection with only one dosage [170]. In an investigation, a nanovaccine konown as SCTV01B is rationally produced by utilizing the RBD of the COVID-19 virus S protein in combination with the capsular polysaccharide of Streptococcus pneumoniae serotype 14 (PPS14) as the backbone. The ultimate formulation of interlaced NPs in the network construction shows great thermal consistency. Immunization with this nanovaccine stimulates strong humoral and Th1/Th2 cellular immune reactions in animal models. Especially, SCTV01B-immunized serum not only widely cross‐neutralizes whole COVID-19 virus variants of concern (VOCs), such as the novel Omicron variant but also demonstrate great opsonophagocytic activity (OPA) versus S. pneumoniae serotype 14. Generally, these encouraging preclinical outcomes support better clinical assessment of SCTV01B, determining the capability of polysaccharide‐RBD‐interlaced NP vaccine methods for the progress of vaccines for SARS-CoV-2 and other infectious diseases [171]. GBP510 is a recombinant protein vaccine that contains self-assembling, two-ingredient NPs showing the RBD of the S protein in an extremely immunogenic array. Dissimilar common protein subunit vaccines, which are decomposed via different proteases in the blood, GBP510 is engineered to exhibit COVID-19 RBD on NP scaffolds. Therefore, it could stimulate potent antibody reactions with postponed destruction. Furthermore, GBP510 can supply preservative immunity versus various evasion mutants via stimulating antibodies targeting several protected, non-overlapping RBD epitopes. Consequently, in an animal model, vaccination with RBD NPs induced 10-fold greater neutralizing antibody titers contrasted to the perfusion-stabilized S ectodomain trimer, even at a five-fold lesser dosage, and preserved versus mouse-adapted COVID-19 virus challenge. Multivalent RBD nanovaccine induced various polyclonal antibody responses, affording protection against heterosubtypic COVID-19 virus [172]. The immunogenicity and efficacy of a COVID-19 vaccine comprising stabilized, pre-fusion S protein trimers were demonstrated in a different investigation using ferritin NP (SpFN) adjuvanted with either conventional aluminum hydroxide or the Army Liposomal Formulization QS-21 (ALFQ) in an animal model. Vaccination led to strong cell-interceded and humoral reactions and a remarkable decrease in lung damage after COVID-19. The power of the immune reaction proposes that dosage sparing via decreased or one dosing in primates may be potential with this vaccine. In general, the data contribute to a more accurate evaluation of SpFN as a COVID-19 viral protein-based vaccination candidate when taking schedule and fractional doses into account [173].

## Application of nanoparticles in diagnosis

**Fig. 4:**
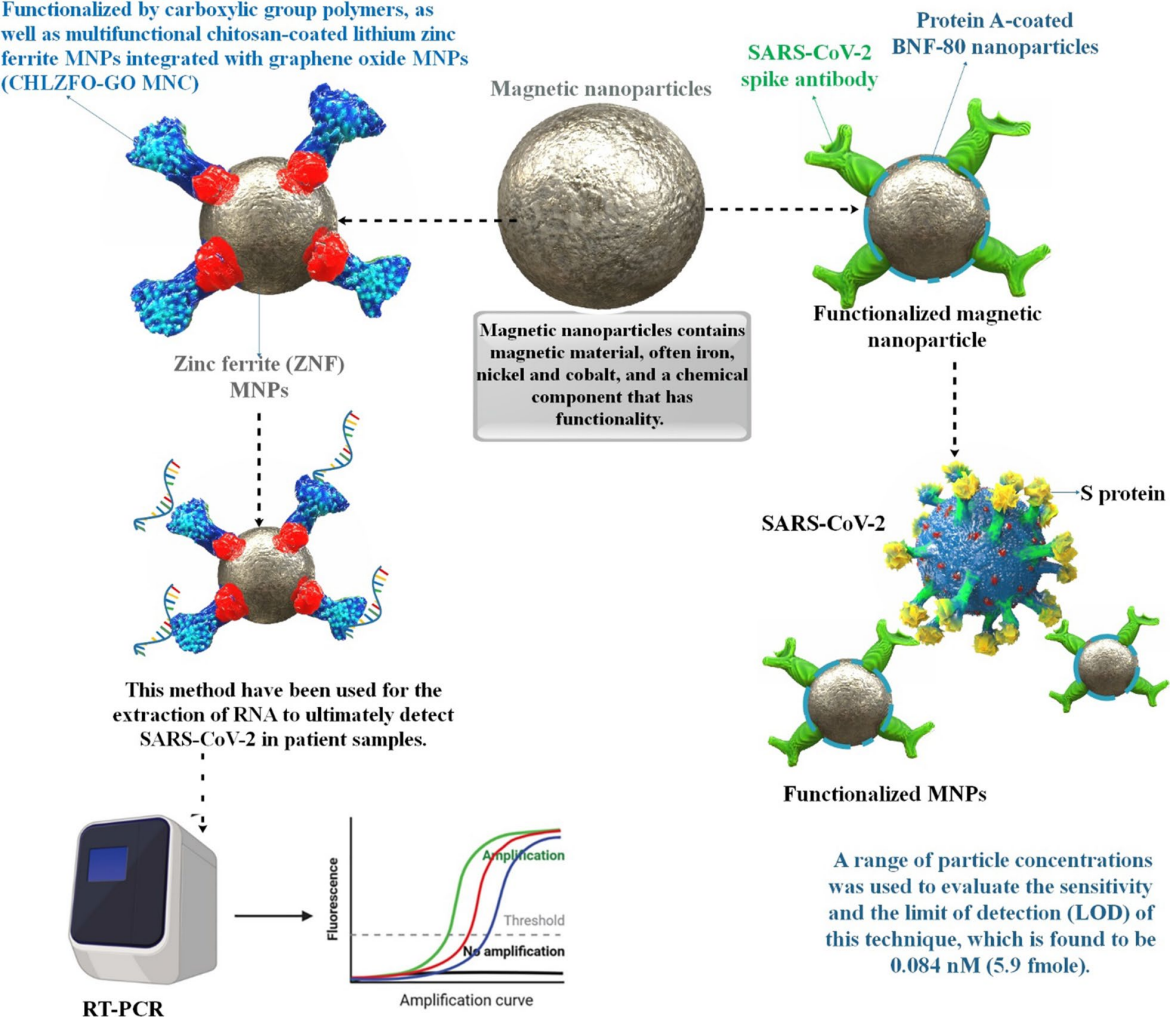
The application of MNPs in the detection of the SARS-CoV-2 virus. The novel progresses in MNPs and nanotechnologies have changed standard diagnostic techniques to nanoscale and pushed the limit of detection to early-stage COVID-19 detection. Generally, MNPs are functionalized with antibodies or DNA/RNA probes that can particularly connect to target analytes

According to the Centers for Disease Control (CDC), the SARS-CoV-2 genome sequence should be screened for infectious agents. With a nasopharyngeal sample, reverse transcription and the synthesis of complementary DNA (cDNA) may be utilized to detect SARS-CoV-2 in the body. [174]. This approach entails shipping patient specimens to a research institution for analysis, which might take a few days. Due to the time-consuming nature of this method, the magnetic properties of NPs (for example, gold) can be used for the rapid detection of CoV [121]. Consider the following approach to diagnosing the magnetic properties of SARS-CoV-2: In these laboratory experiments, a membrane strip with two lines is coated: one line displays AuNP-antibody conjugates, while the other line traps antibodies. Capillary action pulls the proteins across the strip once the patient’s sample is introduced to the membrane. Antigens bind to antigen-antibody conjugates made of AuNPs as they pass through the first line, and the complex travels through the membrane together. The complex system is immobilized by trapping antibodies as they reach the second line, resulting in a red or blue line indicating the presence of the virus. The red line represents individual AuNPs, whereas the blue line represents clustered AuNPs. This approach might be employed to decrease the detection time for viruses [23, 122]. Early and quick identification is critical to reducing the disease’s prevalence. Biosensors based on NPs can precisely detect biomarkers such as nucleic acids (DNA or RNA), specific antigens (proteins or enzymes), or antibodies to detect SARS-CoV-2 [175–178]. Recent advancements in nanotechnology have enabled the development of a SARS-CoV-2 detection device based on graphene and an anti-spike antibody. No sample preparation or labeling is required for this novel kit, which detects SARS-CoV-2 at deficient levels with great efficiency [179]. In a recent study, a novel non-invasive method for diagnosing SARS-CoV-2 was developed. SARS-CoV-2 antigens trapped in surgical face masks were detected using nanoparticle biosensors. The biosensors show high sensitivity and specificity even when tested on asymptomatic patients wearing masks for only 30 min. A smartphone can interpret the signals, and the entire operation takes less than 10 min, making it suitable for decentralized, point-of-need mass scanning [180]. The COVID-19 outbreak prompted the development of numerous Reverse transcription polymerase chain reaction (RT-PCR) based techniques and kits for detecting SARS-CoV-2 genomic RNA [181, 182]. It has been shown that COVID-19 was detected using RT-PCR. However, its use in viral infection diagnosis and epidemic management is restricted due to the lengthy and arduous methods for processing samples. Nucleic acids are liberated from viral particles before adhering to the column membrane, followed by repeated centrifugation steps to allow binding, cleaning, and elution of the extracted nucleic acids. Techniques based on magnetic NPs have been proven to be easy-to-use, automated, and high-throughput alternatives to centrifugation [47–50]. Traditional Magnetic NPs (MNPs) based methods may selectively absorb nucleic acids from lysed materials due to surface-modified functional groups (Fig. 4). These techniques combine the lysis and binding phases, allowing the carboxyl groups (PC)-coated MNPs-RNA (pcMNPs-RNA) complexes to be introduced to future RT-PCR studies soon after they have been formed. Using this streamlined methodology, simple manual methods or automated high-throughput technologies may isolate viral RNA from multiple samples in 20 min or less. With this new extraction method, COVID-19 molecular diagnostics, especially for early clinical diagnosis, might be considerably shortened in terms of turnaround time and operational needs [49]. Using SARS-CoV-2 nucleoproteins modified with selenium NPs, researchers have developed a lateral flow immunoassay kit that detects anti-SARS-CoV-2 IgM and IgG in human blood under 10 min. According to their findings, the selenium nanoparticle lateral flow test can detect anti-SARS-CoV-2 IgM and IgG in human serum and blood quickly and accurately, which it can be used in COVID-19 epidemiological studies [183]. A recent research used a 5-minute colorimetric change observation to construct anti-spike antibody-attached AuNPs for the rapid detection of specific COVID-19 viral antigens. Surface-enhanced Raman spectroscopy (SERS) was used to identify the 4-amino thiophenol-gold (as a reporter molecule) nanoparticle Au–S bond for quick and sensitive identification. The AuNPs agglomerate as soon as COVID-19 antigen or virus particles are present, altering their color from pink to blue, allowing the presence of antigen or virus to be detected immediately by the naked eye. Even at concentrations as low as one nanogram (ng) per mL for COVID-19 antigen and 1000 virus particles per mL for SARS-CoV-2 spike. Significantly, the accumulated AuNPs formation “hot spots” to supply very powerful SERS signal amplification from anti-spike antibody and 4-amino thiophenol-connected AuNPs via light-matter interplays. Finite-difference time-domain (FDTD) simulation information shows a four folds-of-size Raman amplification in “hot spot” situations when AuNP formation accumulates. The results show that antibody and 4-amino thiophenol connected AuNPs-based SERS probe can diagnose SARS-CoV-2 antigen even at a density of 4 picograms (pg) per mL and virus at a density of 18 virus particles per mL during a 5-min time frame [57]. Rapid and precise diagnostic techniques for SARS-CoV-2 diagnosis, treatment, and infection management are critically needed. A one-step reverse transcription loop-mediated isothermal amplification (RT-LAMP) assay employing NPs and RT-LAMP was successfully developed for the quick and reliable detection of COVID-19. Basic equipment such as a heater block was required to maintain a steady temperature (63 °C) for just 40 min. Two specially designed LAMP primer sets were used to identify SARS-CoV-2 F1ab and nucleoprotein genes. The detection results were verified by NPs-based biosensors (NBS). To identify SARS-CoV-2 infection in the first line, public health, and clinical laboratories in resource-limited locations, RT-LAMP coupled with NBS assay (RT-LAMP-NBS) might be used [184]. To detect COVID-19 quickly, sensitively, and specifically and developed a reverse transcription multiple cross-displacement amplification (RT-MCDA) biosensor assays using NPs (RT-MCDA-BS). The open reading frame (ORF 1a/b) and nucleoprotein gene of SARS-CoV-2 were targeted using two primer sets. The reaction was maintained at 64 °C for 35 min using a simple setup. The COVID-19 RT-MCDA-BS test method canm; be completed successfully in about an hour. The COVID-19 RT-MCDA-BS can identify the target sequence with only five copies [185]. For detecting COVID-19, combined a multiplex RT-LAMP (mRT-LAMP) assay with a nanoparticle-based lateral flow biosensor (LFB) assay and mRT-LAMP-LFB was created. The SARS-CoV-2 ORF1ab and nucleoprotein genes were amplified in a single-tube reaction using two LAMP primer sets, and the diagnostic results were simply analyzed by LFB. The full diagnostic exam, including sampling and analysis of data, may be performed in about an hour. In conclusion, the COVID-19 mRT-LAMP-LFB test is one of the potential methods for detecting agents of COVID-19 disease in laboratories, particularly in resource-poor countries’ laboratories [186]. Drug therapy by the assistant of NPs Viral infections bind to receptors in host cells by infectious agent molecules, which then enable virus entry. SARS-CoV-2 cell binding and induction are aided by the S-spike glycoprotein [187, 188].

Consequently, halting viral endocytosis or interference with the virus’s capability to bind to the ACE2 receptor could be a potential therapeutic study and treatment strategy. Chloroquine is a drug that was examined to investigate the above process. Chloroquine has been demonstrated to block NP endocytosis; since SARS-CoV-2 particles physically resemble NPs, chloroquine can prevent SARS-CoV-2 particles endocytosis. The postulated mechanism involves a chloroquine-induced reduction in PICALM (phosphatidylinositol binding clathrin assembly protein) restricts NPs, including SARS-CoV-2, are absorbed by endocytosis. Chloroquine effectiveness is governed by its transport and cellular absorption, both of which can be considerably improved by encapsulating the medication in polymeric NPs. Polylactic acid (PLA) polymeric NPs are the most often used for encapsulating chloroquine [46]. In addition to assisting drug delivery, NPs may directly interfere with viral receptor binding and cell entry. Found that cationic carbon dots (CCM-CDs) produced from curcumin with a diameter of 1.6 nm can prevent the viral access of the pig epidemic diarrhea virus (PEDV), a CoV model [189].

The foremost challenge is an absence of efficient therapy for the COVID-19 threat. Nevertheless, it has been treated with various combinations of antiviral drugs worldwide, and many additional potential therapies are being studied under clinical trials. However, RDV has demonstrated considerable results on SARS-CoV-2, and it is the only FDA-approved therapy for CoV in the United States. [190]. Furthermore, chloroquine and hydroxychloroquine had no significant influence on improving patients’ clinical outcomes, and the confidence of the evidence was inconsistent among investigations [190–193]. The combination of lopinavir and ritonavir, as well as their combination with other agents, has not yet yielded encouraging results in the treatment of COVID-19 [194–196]. However, in China, studies report that Favipiravir is more effective with no serious adverse effects [197, 198]. Avifavir, antiviral medication, and Hydroxychloroquine are being distributed in Russia [199]. In another study, molecularly imprinted polymer NPs (nanoMIPs) are prepared with a rapid (2 h) and scalable procedure utilizing merely a small COVID-19 virus particle (about ten amino acids). The nanoMIPs match the affinity of COVID-19 virus antibodies under legal trial situations and excel at increased temperatures or in an acidic environment. Thus, the nanoMIP sensors method has apparent benefits over antibody-based methods as they can function in different challenging environments. Heat transfer-based evaluations show better limits of detection contrasted to mercantile fast antigen analyses and most antigen analyses from the literature for alpha (∼9.9 fg mL^–1^) and delta (∼6.1 fg mL^–1^) variants of the S protein. A prototype evaluation is produced, which can quickly (about 15 min) confirm clinical patient samples with well accurately and selectively [200]. The conjunction of imprinted particles into a micelle-based electrochemical cell manufactured a signal when combined with particle analytics (including the COVID-19 virus) formerly imprinted onto the construction. Nanoamp scales of motions were produced from what may have sensitively been unique virus-micelle interplay. The method demonstrated sensitivity when evaluated versus similar dimensions and morphology particles. The procedure was suitable for airborne aerosol sampling methods. Generally, the imprinted micelle method could supply near real-time diagnosis techniques to various potential analytics of interest in the field [201].

## Are AgNPs a way to get rid of Covid-19?

COVID-19 is a highly infectious and ubiquitous illness that has put tremendous pressure on the world’s healthcare system. The antimicrobial, antiviral, immunomodulatory, and biosensing activities of AgNPs are well recognized. AgNPs have been discovered to be possible antiviral drugs that work against various dangerous viruses, including COVID-19. AgNPs may produce free radicals and ROS, which cause apoptosis and therefore prevent viral infection. AgNPs’ form and size are essential in their biomedical applications since changes may lead to different biological interactions and activities. By emphasizing the present state of AgNPs in the battle against COVID-19, we suggest that they may be used to manage the continuing COVID-19 pandemic effectively [41]. Metals like calcium, iron, selenium, and zinc are required for development and survival in a variety of metabolic processes [202–204]. NPs are well-known for various benefits, including enhanced surface area, customized cargo release profiles, drug pharmacokinetics modification, decreased toxicity, and better biological response [29, 205]. AgNPs are considered one of the most promising therapeutic NPs due to their unique catalytic, optical, and clinical applications and are incorporated into most commercialized NPs. AgNPs may trigger an immune response in host cells and cause inflammatory cell death. Many Ayurvedic medicines, notably enriched Chyavanprash and Bhasmas, include silver (fine.

metal powders that are said to be nano-sized). The silver Bhasma has been used for centuries to cure inflammation, discomfort, memory loss, and other ailments. Silver has also been found to help with several inflammatories, cardiovascular, and other non-communicable diseases [206–209]. AgNPs have potent antiviral effects, making them a good candidate for vaccine development. These NPs attach to the viral genome and inhibit the activity and interaction of various viral and cellular replication components, resulting in viral replication suppression and the release of progeny virions [40]. Lung inflammation and fibrosis are joint in COVID-19, which is also linked to lung fibrosis[210–212]. AgNPs may be a potential therapeutic alternative with anti-inflammatory and anti-fibrotic properties because they inhibit inflammatory cytokines by modulating their transcriptional activity. The Nuclear factor kappa B-cells (NF*-*κB) and Mitogen-activated protein Kinase (MAPKs) pathways were modulated by AgNPs in preclinical animal models, leading to reduced inflammation and decreased fibrotic cascades. In addition, IL-1B, IL-6, IL-17, TGF-β, and TNF-α levels were also reduced by AgNPs in preclinical animal models [39, 213–216]. In a recent study, AgNPs were found to exhibit anti-fibrotic properties in an induced colitis model utilizing dextran sodium sulfate (DSS). AgNPs reduced collagen deposition by lowering the expression of profibrotic genes such as Col lal and Col la2, as evidenced by histological findings confirmed by mRNA expression analysis. Therefore, AgNPs may prevent COVID-19 inflammation and fibrosis by reducing the amounts of cytokines and cytokine-mediated inflammation in the body [42]. Because of their powerful antimicrobial properties, AgNPs offer a novel way to limit the SARS-CoV-2 virus’s development while preventing secondary microbial infections. AgNPs with a size range of 2–15 nm exhibit antiviral properties against SARS-CoV-2. Furthermore, AgNPs may inhibit cytokine storm, inflammatory signaling, pulmonary insufficiency reduction, and regulation of the EMT signaling cascade. AgNPs provide a variety of options for developing extremely sensitive biosensors. We expect that AgNPs will play a critical role in the fight against the COVID-19 epidemic in the not-too-distant future [38]. Based on recent findings, researchers using a new LNP delivery method report a very efficient siRNA therapy against SARS-CoV-2 infection. Multiple siRNAs targeting highly conserved areas of the SARS-CoV-2 virus were tested, and three candidate siRNAs emerged that efficiently suppressed the virus by more than 90% alone or in combination. They created and tested two new LNP formulations for delivering potential siRNA treatments to the lungs, an organ that suffers significant damage during SARS-CoV-2 infection. The in vivo injection of siRNAs encapsulated in these LNPs resulted in viral suppression in the lungs and a significant survival benefit for the treated mice. Their LNP-SIRNA procedures may be measured or evaluated on a scale and may be recommended for patients as soon as they exhibit COVID-19 symptoms. We believe that a siRNA-LNP therapeutic strategy could be highly beneficial in treating SARS-CoV-2 infection as an additional security treatment to existing immunization methods [26].

## Conclusion

COVID-19 has had a detrimental impact on people all across the world, but because of extensive immunization, the disease’s prevalence and severity have diminished. As a result, preventive materials, early diagnosis, and treatment are critical. Nanotechnology and NPs offer a lot of potential for reducing the threat of COVID-19. To combat COVID-19, NPs must be used strategically in a variety of fields, including biosensors, spraying, coating, medication delivery, and therapeutics. NPs have advantages attributed to their sizes such as multifold functions in different particle dimensions, including surface modification, targeted medication delivery, carrying several types of antiviral agents simultaneously, bio-adaptability, gradual and continuous medication discharge, which endows them with great potential in the field of disease treatment and prevention. In addition, NPs can improve the targeted delivery of available antiviral medications through long-lasting blood circulation time, connection to target cells, and facilitating bioavailability. NPs can significantly increase the yield of lipophilic medicines, which are usually difficult to manufacture and deliver in vivo. Because of NPs ideal size characteristics and high surface area, NPs can be dependent on viruses in a multivalent technique, permitting extraordinarily more binding strong interactions. Furthermore, the NPs are used as curative agents, because of their minor side effects, greater specificity, and immune-modulatory properties, can generate significant efficacy in combating the SARS-CoV-2 infection. Moreover, several NPs-based formulations are on the marketplace for COVID-19 prevention (hand disinfectants, masks, etc.). Different NPs, such as AuNPs, enable quick and efficient COVID-19 detection and can be used for SARS-CoV-2 infection management and control. As the preponderance of COVID-19 is faster than the development of practical prevention tools, detection methods, vaccines, and antiviral drug investigations should be supplementary to what has already been achieved with previous SARS-CoV-2 dependent investigations.

## Data Availability

Not applicable.

## Abbreviations

SARS-CoV-2: Severe acute respiratory syndrome coronavirus 2
COVID-19: Coronavirus disease 2019
CoV: Coronavirus
NPs: Nanoparticles
N: Nucleocapsid
M: Membrane
E: Envelope
S: Spike
ACE2: Angiotensin-converting enzyme 2
NTD: N-terminal domain
RBD: Receptor-binding domain
AuNPs: Gold nanoparticles
AgNPs: Silver nanoparticles
IONPs: Iron oxide NPs
CuNPs: Copper nanoparticles
MNPs: Magnetic nanoparticles
LNPs: Lipid nanoparticles
GO: Graphene oxide
IL: Interleukin
TGF-β: Transforming growth factor-beta
TNF-α: Tumor necrosis factor alpha
MD: Molecular dynamics
Mes: 3-Mercaptoethylsulfonate
Mus: Mercapto undecanol sulfonic acid
Ot: Octanethiol
FETs: Field-effect transistors
CNTs: Carbon nanotubes
TiO: Titanium dioxide
PPE: Polyphenylene ethynylene
EMT: Epithelial-mesenchymal transition
CRS: Cytokine release syndrome
HA: Hyaluronic acid
PNAB: PEGylated nanoparticle albumin-bound
EM: Transmit electromagnetic
NagC: Nano-silver colloids
IC: Inhibitory concentration
MIC: Minimum inhibitory concentration
VAP: Ventilator-associated pneumonia
WHO: World Health Organization
EUL: Emergency use listing
EID: Emerging infectious diseases
CTL: Cytotoxic T lymphocyte
FDA: Food and Drug Administration
HIV: Human immunodeficiency virus
CMV: Cytomegalovirus
HPV: Human papillomavirus
HBV: Hepatitis B virus
CDC: Centers for Disease Control
cDNA: Complementary DNA
RT-PCR: Reverse transcription polymerase chain reaction
SERS: Surface-enhanced raman spectroscopy
Ng: Nanogram
FDTD: Finite-difference time-domain
Pg: Picograms
RT-LAMP: Reverse transcription loop-mediated isothermal amplification
NBS: NPs-based biosensors
RT-MCDA: Reverse transcription multiple cross-displacement amplification
ORF: Open reading frame
mRT-LAMP: Multiplex RT-LAMP
LFB: Lateral flow biosensor
PICALM: Phosphatidylinositol binding clathrin assembly protein
PLA: Poly lactic acid
CCM-CDs: Cationic carbon dots
Mφ: Macrophage
PEDV: Pig epidemic diarrhea virus
ROS: Reactive oxygen species
NF*-*κB: Nuclear factor kappa B-cells
MAPKs: Mitogen-activated protein Kinase
siRNA: Short interfering RNA
